# Tackling standardization in clinical research workforce hiring using competency-based job classifications

**DOI:** 10.1017/cts.2023.672

**Published:** 2023-11-06

**Authors:** Christine Deeter, Deborah Hannah, Marissa Stroo, Rebecca Brouwer, Heather Gaudaur, Stephanie A. Freel, Denise C. Snyder

**Affiliations:** 1 Duke Office of Clinical Research, Duke University, Durham, NC, USA; 2 School of Medicine, Duke University, Durham, NC, USA; 3 Clinical and Translational Science Institute, Duke University, Durham, NC, USA; 4 Duke Office of Research Initiatives, Duke University, Durham, NC, USA; 5 Duke Human Resources, Duke University, Durham, NC, USA

**Keywords:** Clinical research, hiring, competencies, workforce development, career pathways

## Abstract

In 2016, Duke reconfigured its clinical research job descriptions and workforce to be competency-based, modeled around the Joint Taskforce for Clinical Trial Competency framework. To ensure consistency in job classification amongst new hires in the clinical research workforce, Duke subsequently implemented a Title Picker tool. The tool compares the research unit’s description of job responsibility needs against those standardized job descriptions used to map incumbents in 2016. Duke worked with human resources and evaluated the impact on their process as well as on the broader community of staff who hire clinical research professionals. Implementation of the tool has enabled Duke to create consistent job classifications for its workforce and better understand who composes the clinical research professional workforce. This tool has provided valuable workforce metrics, such as attrition, hiring, etc., and strengthened our collaboration with Human Resources.

## Introduction

The clinical research landscape is increasingly complex and has changed considerably in the past twenty years, requiring upskilling of the clinical research workforce [1–5]. Significant work has taken place to define clinical research job competencies [6–10]. Today, the Joint Taskforce for Clinical Trial Competency (JTFCTC) framework is used by Academic Medical Centers (AMCs), pharmaceutical industry, and Clinical Research Organizations alike and is available in seven languages [11]. Despite this carefully crafted framework, alignment of current positions with competency profiles is not simple, requiring reworking of individual job roles and an entire workforce model [8,12]. Duke pioneered the adoption of the JTFCTC framework at an AMC to establish a competency-based workforce model by coalescing more than 80 titles into 12 standardized, well-defined job classifications [9]. These classifications established career ladders and created a built-in professional pathway by incorporating tiered positions through which employees can advance [10]. To maintain this standardization across Clinical Research Professional (CRP) positions, a tool, called the Title Picker, was developed in Research Electronic Data Capture (REDCap) [13] to systematize job postings aligned with clinical research competencies across the enterprise.

Currently, the Duke clinical research workforce totals more than 900 CRPs, with approximately 200 new hires each year. To ensure that each CRP hire or promotion falls under the appropriate job classification, the hiring manager must complete a Title Picker survey prior to finalizing any CRP job description and posting. Using this methodology, Duke has maintained standardization of clinical research professional jobs across the workforce--setting competency-based levels for each position, and most critically, establishing internal functional alignment by job code across our CRP workforce.

## Materials and Method

### Ideation of the Title Picker Tool

In 2016, the Clinical Research Professionals Working Group (CRPWG) at Duke developed an initial tool in REDCap to map incumbent CRPs into the 12 new clinical research job classifications. This tool utilized the clinical research competency framework, and our recently-developed competency leveling (described in Brouwer *et al*., 2017), to detail existing job responsibilities for our current clinical research staff [9]. The job mapping tool incorporated a competency level scale (examples provided in Brouwer *et al*. 2017) based on levels of responsibility, complexity of task, oversight and mentorship, and independence assigned to each job task aligned with the JTFCTC competency framework [6,9]. For example, in this scale, which ranged from 1–5 with respect to increasing complexity, the number 1 level designated responsibilities expected and assigned to entry-level positions. Number 5 levels correlated to competency-based activities requiring more complex skills and independence as expected for those in higher-level leadership positions. Some competency areas or tasks require autonomy and are not appropriate at the entry levels; these tasks would be excluded from lower-level options within the mapping tool [9]. This mapping process was instrumental in the development of the new clinical research (CR) competency-aligned framework for current CRPs and future hires.

As we developed this tool for mapping incumbent competency levels and respective job titles, we discussed methods for maintaining an equitable job landscape as new CRPs were hired. Prior to the job mapping in 2016, our CRP hiring process, much like those across AMCs, depended on departmentally siloed hiring managers (and often, faculty) making somewhat arbitrary decisions about which existing job title to post. Oftentimes these decisions were influenced more by budgetary considerations or comparisons with the performance of current employees than by the task portfolio or level of responsibility required for the job. At the same time, a dispersed CR ecosystem made it difficult for hiring managers to visualize and comprehend how job titles are incorporated in different areas, creating the perfect storm for job inequity across the workforce [1,14]. The competency mapping tool provided an ideal platform for defining CRP job requirements during the hiring process. Reconfigured as the Title Picker, this tool has been key for standardizing jobs within our CRP workforce and for maintaining consistent job expectations and responsibilities among incumbents and new hires. While the mapping of incumbents into the new positions went into effect in September 2016, new CRPs hired on or after July 2016 were hired into the newly established job classifications using the Title Picker tool. This implementation timeline was critical to avoid reverting to prior hiring methods. While it appears that new hires were first, the processes were taking place concurrently. The previous job descriptions were delimited and replaced with the new job classifications, allowing managers to hire into the new positions rather than perpetually hiring into the old classifications, which would necessitate mapping additional incumbents.

The Title Picker tool was developed out of the initial mapping tool in REDCap. The tool was reconfigured from asking what incumbents were responsible for in their current jobs to asking what tasks and levels of responsibility a hiring manager needed a new hire to perform. This critical shift focused the job code selection specifically on the business needs for the new position prior to posting and less on subjective measures.

### Title Picker Procedure

The Title Picker survey is built in REDCap and completed by the hiring manager. This process was set up, and operationally supported, by the Clinical Research Professionals Workforce Group (CRPWG). CRPWG was a multidisciplinary working group established to encompass expertise from clinical research across various disciplines and human resources [9]. This group evolved in 2017 to establish a program at Duke known as Workforce Engagement and Resilience program (WE-R). Today, WE-R encompasses hiring, training, onboarding, professional development, pathways into the clinical research profession, and data collection and analysis related to the clinical research community at Duke.

**Figure 1.**
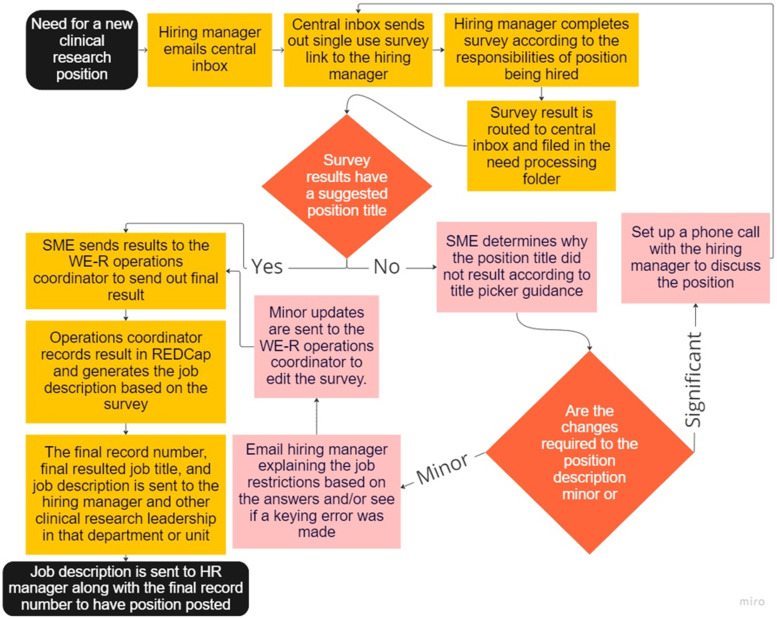
Title Picker process workflow; workforce engagement and resilience (WE-R) subject matter expert (SME) research electronic data capture (REDCap).

To begin the Title Picker process, the hiring manager sends a request to a central email address, managed by the WE-R operational team. The WE-R team then sends the hiring manager a single-use link to the Title Picker form. Title Picker results are calculated automatically within REDCap using a responsibility scale described above, with each job code determined by an algorithm, considering both the competency profile selected and the levels of complexity and responsibility for each specific task. Survey results are routed to a subject matter expert (SME) from the WE-R team to review and assign the job title based on the calculated results and any comments included by the hiring manager. In some instances, the selections on the survey do not clearly map into a position. In these cases, the WE-R team works with the hiring manager to better understand the role and expectations, aids the manager in redefining the position needs, and assists in resubmission as needed. The results, including corresponding title and general job description, are then sent from the central WE-R email address to the hiring manager and other departmental clinical research leadership for posting (Fig. 1).

### Implementation Considerations

At first, the Title Picker process for job creation was challenging to implement. The clinical research managers mapped 700 existing staff using the mapping tool, which required managers to respond to activities that their staff were already doing. In contrast, the Title Picker requires managers to anticipate job responsibilities needed for a planned position. As explained in more detail below, the Title Picker is also used for re-classifications. If a position is already filled and the scope warrants a change in classification, a new submission would need to be generated. To alleviate confusion, we provided small group, hands-on training to managers and presented to the Clinical Research Unit manager meeting that represents 22 units aligned with our clinical departments, centers, and institutes. The training focused on selecting the activities and competency level needed for the job. This was an important distinction to ensure managers were not selecting a job based on the position title or budgetary constraints. Early on, a great deal of time was spent with managers to clarify the process and re-orient to the principles of the equity- and competency-based framework.

A website hosts guidelines of requirements related to posting or reclassifying any of the 12 CRP job classifications (https://medschool.duke.edu/research/research-support/research-support-offices/duke-office-clinical-research-docr/workforce-5). To ensure consistent use of this process, we partnered with HR leaders to put fail-safes in place. All requests for newly-posted positions or job re-classifications must be assigned a Title Picker record number prior to HR approval and posting. Compensation, responsible for job classification for the enterprise, reviews the submission to ensure this record number is present. If the record number is missing, the hiring manager is directed to submit a Title Picker to the WE-R team. Furthermore, many of our clinical research systems (e.g., the electronic health record) have access limitations based on job title or role. When new access requests are made, job codes are reviewed before access is granted through the central office. This ensures consistent use of the Title Picker and minimizes hiring into non-CRP if the position has clinical research responsibilities.

The Title Picker review process and job code assignment were initially manual. In the first version of the Title Picker tool, submitted forms were manually exported into a CSV file and mail merged to develop a position profile with a record-specific job description. The numerical competency levels were taken from the Title Picker results to develop the job position profile. The profile was sent to an SME to manually compare the output to a general guidance that was developed out of the mapping process. If there was a discrepancy from the pre-defined criteria, the reviewer would call the manager to discuss and request updates to the Title Picker form to ensure alignment. With the initial release of the Title Picker, this happened frequently; over time, managers gained a better understanding of the competencies themselves and the number of these conversations decreased. While specific tasks and competencies within the job profile could differ (e.g., specific therapeutic areas may have international trials while others may not), tasks were leveled in relation to each other to ensure consistency in expectations and responsibility level for each job across Clinical Research Units.

As the number of Title Picker submissions increased, we moved to an algorithmic approach to derive outputs. We used past submissions to identify competencies and levels that were specific to each position. This led to a calculation that provided a “best fit” title based on the unique combination of Title Picker answers. The algorithm used averages of competency levels (1–5) to calculate the overall leveling of the position. Responses regarding the type of clinical research being conducted and competency level of the tasks required for the role further ensured that positions had accurate job responsibilities at the appropriate level. These notations may also be included in the job posting to specify special skills or experience. Below are some examples of major factors that dictate how a position should be classified.

- A Regulatory Coordinator is not expected to perform recruitment, consenting, or patient-facing activities. Thus, the definition of the Regulatory Coordinator job code includes the absence or low level of responsibilities in these competency areas.
- Jobs requiring clinical responsibilities typical of a licensed nurse are designated the Clinical Research Nursing Coordinator (CRNC) job code. The CRNC role requires specific licensing and health system practice credentialing. However, RN candidates may fill other CRP jobs if the position does not require use of their nursing skills in the job.
- The entry-level positions, CRS and CRS Sr., which are expected to have low levels of independence, have restrictions on medical record access, documentation, preparing orders for physician sign off and release, and complexity of the studies and tasks to which they are assigned.

Additional questions are included in the Title Picker survey to assess roles with more complex or unique responsibilities, such as those found in the Research Program Leader position. When portfolio or program management responsibilities are indicated in the Title Picker form, the tool requests more detailed explanation of the breadth of the research portfolio to be managed, along with an organizational chart. In these cases, the Associate Dean of Clinical Research reviews and approves the Title Picker form to ensure appropriate organizational need for these high-level positions prior to communication of the result to the submitter.

### Assessing Institutional Impact on Human Resources

Survey interviews were conducted by an external member of the WE-R group of seven of our human resources (HR) staff, including Compensation (3), Departmental HR (3), and School of Medicine HR (1). Participants were asked a series of open-ended questions about whether they were working during the implementation of the Title Picker process, if their role included clinical research jobs that fall under the Title Picker purview, how the Title Picker process impacts their work, and what they would say to HR or Compensation managers and leaders at other institutions who are considering a similar Title Picker implementation.

Three themes that stood out during the interviews with HR were consistency, documentation, and equity. Staff appreciated efforts to streamline position determination and offer an objective review mechanism to determine the appropriate job level, as this function is performed informally for other job classifications, through email and telephone. Most recommended the process for other institutions, however, they acknowledged that there is a heavy lift to create the tool, train, support, and obtain buy-in across the institution. One interviewee highlighted the previous struggle in clinical research hiring, prior to the implementation of the Title Picker, was related to inconsistent documentation across units. The Title Picker process provides an opportunity to capture data in a structured manner, consistently across all groups as described by this interviewee.

*“The same questions are being asked, the same information is being provided, the same group [is reviewing the results], the same tool is [being used to assess data]; so, I think it really allows you to make consistent decisions. Not everybody is always going to agree with those decisions. Obviously, when people come to our groups and want a position classified, or they're looking for a certain level or a certain title. They probably have something in their mind of what they're seeking, but I think we can confidently answer when they go through this tool that we’re being consistent.”*

## Results

**Figure 2.**
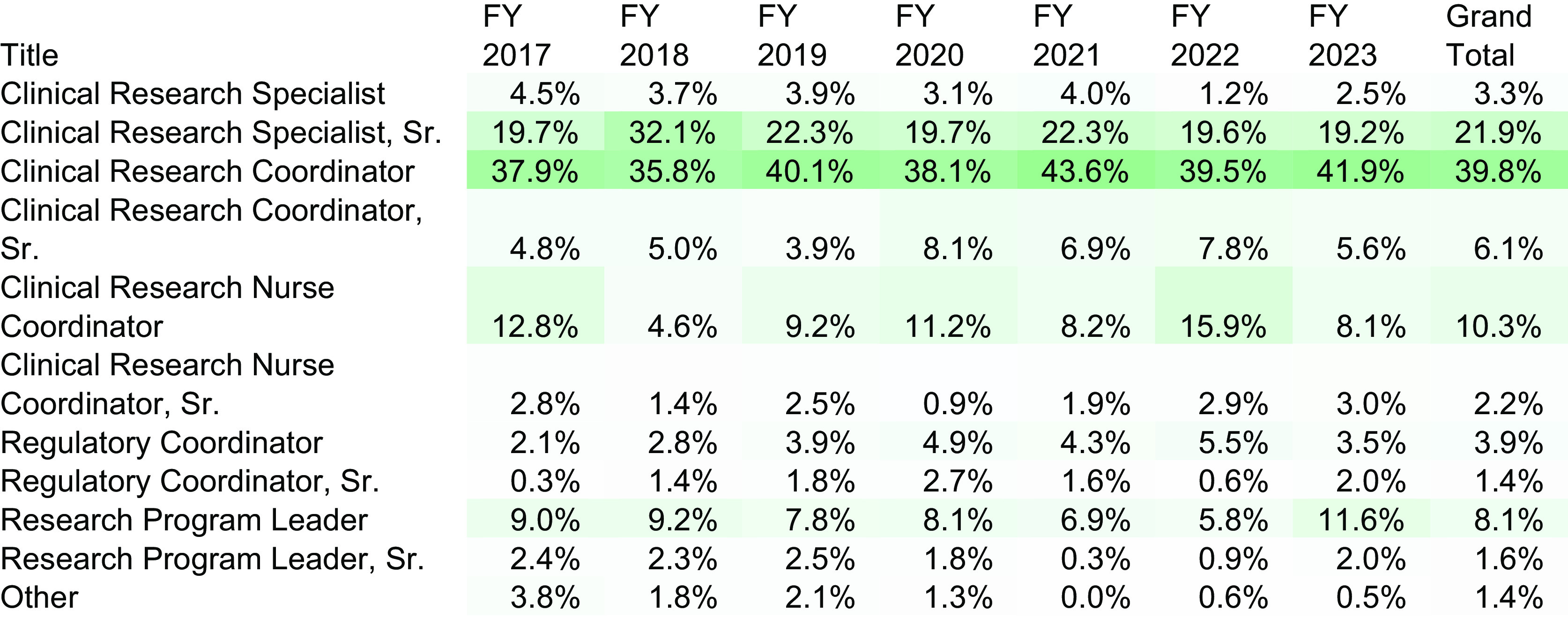
Title Picker submissions and resulting returned (*N* = 1934); senior (Sr.).

Since its inception, 1,934 Title Pickers have been submitted to open or reclassify a position and given a proper suggested title or job code. Fiscal year 2016 was excluded from this data as *N* = 3 for this time period. Fig. 2 displays the percentages of each position that make up the total number of Title Pickers completed for each fiscal year. Consistently, the bulk of our workforce is made up of the salaried Clinical Research Coordinator and the hourly Clinical Research Specialist Sr. (together comprising more than 60% of staffing). We state that results will be sent to the department within 5–7 business days of completion. However, when analyzing the difference between survey completion (date hiring manager completed the Title Picker) and sending the title picker result (date department was provided a Title Picker result), the average is 3.1 days. In this calculation, 20 records were excluded due to lack of survey time stamp or contact date. Our data does not lend itself to determine the lapse between when a result is sent to the department to when the position is posted. This may vary for many reasons including, start of funding, study start up, department workload, etc.

Using a centralized, systematic position determination process: (1) our workforce has maintained alignment with our competency-based framework, (2) we can readily report attrition, hiring, and other metrics (e.g., demographics), (3) and we can design and target training for Duke’s clinical research professional workforce. As reported by Stroo et al., 2020, WE-R has positively impacted attrition with a 3-year, 30% reduction post mapping compared with 3-year attrition rates prior to mapping to the new competency-based jobs [15]. Ongoing internal analyses suggest improvements have been sustained post-pandemic for both attrition and turbulence.

Our WE-R team, as part of the mission of the Duke Clinical and Translational Sciences Award, Participant and Clinical Interactions Core, has consulted with many AMCs to share tools, strategies, and lessons learned from implementing standardized job classifications and the Title Picker tool. Our website is publicly facing with standard job descriptions and tools. Specifically shared on our website are the tools for implementation including the data dictionary for our initial iteration of the Title Picker process, how to generate the job descriptions out of REDCap, a sample summary report as reviewed by our SMEs, and a basic implementation guide. Through our collaborations, the University of Alabama Birmingham adopted a similar process for their clinical research professionals after mapping their incumbents in 2020 [16]. We have consulted with 25 additional AMCs and clinical research sites interested in standardizing their job definitions and hiring processes.

## Reflections

The Title Picker is required for all positions being hired into a WE-R job description. This requirement encourages managers to consider specific job needs, rather than choosing a position based on reasons such as funding or the makeup of their current staff. This process ensures that the manager hires an individual qualified for the responsibilities required of the position rather than defining a job around a specific individual or a desired pay point.

Position selection and posting have improved since implementation of the Title Picker. This is due in part to efficiencies gained in the process, but also, we believe to a culture shift among clinical research professionals at Duke. We aimed to make the benefits of completing the Title Picker greater than the amount of time spent completing it. With each Title Picker result, a position-tailored job description is provided to the hiring manager based on the selections, saving time in drafting job postings. Utilizing the Title Picker tool has guided managers to consider specific job needs, rather than choosing a job title based on subjective criteria. This ensures that a manager can hire a person qualified for the tasks that the job needs, rather than defining the job around specific individual or based on funding.

The Title Picker process has strengthened partnerships with HR practitioners and enterprise leadership. Prior to the Title Picker, the processes for interpreting appropriate positions for hire or reclassification relied on Compensation and HR departmental employees, whose areas of responsibility far exceed the clinical research workforce. With the implementation of the Title Picker, clinical research SMEs can quickly review the position submissions and apply consistent guidance to determine the appropriate position based on the job responsibilities chosen for the positions across research units. This reduced the previous back-and-forth between HR and the hiring manager and lessened the burden on HR staff. In addition, the current process fosters communication between the WE-R SME and the study team or PI to address any concerns about the classification of a new or reclassified position, assisting HR departmental employees.

As previously stated, the Title Picker survey is required to post a new position, to reclassify a filled position, and to fill or reclassify a vacant position. To refill a vacant position, Title Picker results must be no more than one year old. This time frame was designed to address potential business needs and job requirement changes while minimizing burden on short-term re-hiring. While we aimed for a consistent method for each new position, we did not want the burden of completing the Title Picker to be overbearing for hiring managers. To ensure the process did not create unnecessary redundancy, we allowed for up to six new positions to be fulfilled using a single Title Picker survey, as long as responsibilities were equivalent; the positions reported up to the same financial unit; and positions were not re-classifications of existing positions.

Despite the overall acceptance of the Title Picker across HR and Compensation, there are challenges with the tool. While there is standardization, managers and investigators can still “game the system” if they set out to do that. The central WE-R team reviews the managerial or senior positions to ensure consistency in requests, review organizational charts, reiterate the standardization, and seek clarity for any inconsistencies. Downstream checkpoints, as described above, also help to maintain integrity of the process. While more automated and more consistent, these requests require more upfront work from managers to think through what the position needs are for the unit and ultimately, leads to less back-and-forth between HR and compensation (that a Job Analysis Questionnaire often requires).

The Title Picker process has allowed Duke to better manage the clinical research workforce by identifying existing positions across the enterprise, verifying the scope of work for each CRP, and identifying new hires entering the CRP workforce. Another benefit to this hiring process is the ability to target training and onboarding aligned with job competencies for staff hired into WE-R jobs. These distinctive jobs allow Duke to track hiring, attrition, and turbulence across the institution. We can perform employee exit surveys to address employee job satisfaction and attrition. Ultimately, implementation of the Title Picker has been a worthwhile investment by producing consistent, standardized, equitable position selection with accompanying documentation that leads to improved systems control (access matches accountability within job competencies selected). Expectations for these positions are objective and more consistent for both staff and managers. Lastly, although there is an upfront investment in redesigning jobs to meet this competency-based framework, it does streamline job postings for managers, investigators, and HR.
